# Pneumococcal Vaccines: Challenges and Prospects

**DOI:** 10.3390/vaccines7010025

**Published:** 2019-02-27

**Authors:** Paul Licciardi, Ioanna Papadatou

**Affiliations:** 1 Murdoch Children’s Research Institute, Melbourne, VIC 3052, Australia; 2 Department of Paediatrics, University of Melbourne, Parkville, Melbourne, VIC 3010, Australia; 3 Immunobiology and Vaccinology Research Laboratory, First Department of Paediatrics, Aghia Sofia Children’s Hospital, National and Kapodistrian University of Athens, 111527 Athens, Greece

Infections with the bacterium *Streptococcus pneumoniae* are one of the most common causes of morbidity and mortality in children less than five years of age worldwide, mostly in low- and middle-income countries (LMICs). Pneumococcal conjugate vaccines (PCVs) have had a tremendous effect on reducing pneumococcal carriage and invasive disease, due to a combination of direct protection and powerful indirect protection or ‘herd immunity’. Despite the success of PCVs, a number of important issues remain, including the optimal vaccine formulation and vaccination schedule for each setting and for high-risk populations, the impact of serotype replacement following PCV use, transmission of pneumococci in high burden settings as well as understanding the immunological markers of long-term protection against carriage. This special issue features original studies, viewpoints and review articles that aim to address these important issues, and collectively contributing to the optimization of vaccine protection against pneumococcal disease.

In LMICs, mortality due to *Streptococcus pneumoniae* infections among infants and toddlers is exceptionally high; therefore, vaccination strategies aim to offer optimal protection over the first years of life. A study of PCV immunogenicity in Papua New Guinea assessed the immunogenicity and persistence of immunity against pneumococcus induced by two different vaccination schedules in native children. They found that priming with either a 10-valent or a 13-valent PCV at one, two and three months of age, followed by a booster dose of the 23-valent polysaccharide vaccine (PPV23) at nine months of age resulted in protective immune responses in children challenged at 23 months of age [1]. This is an important finding in this setting, where strong immunological priming and broad serotype coverage are needed to protect children at high risk of disease.

In high-income countries, long standing infant PCV vaccination policies have developed strong herd immunity in these populations and have significantly reduced the rates of invasive pneumococcal disease (IPD) among young children. However, the duration of protection induced by these current vaccination schedules remains unclear. A review article by Papadatou et al. summarizes the literature on antigen-specific memory B cells (MBCs) induced by pneumococcal vaccination, and highlights the potential use of this novel marker to monitor the duration of vaccine protection [2]. Circulating antibody titers following vaccination are the only currently accepted in vitro correlate of vaccine protection against IPD but other measures are needed for pneumococcal carriage endpoints. Van Westen et al. examined the waning of serotype-specific antibody after a primary vaccination series with either PCV10 or PCV13 at two, three and four months of age in the Netherlands. Although Immunoglobulin G (IgG) antibody titers were high for the majority of serotypes tested, only serotypes 4 and 19F in the PCV10 group and serotypes 4 and 6B in the PCV13 group reached the protective threshold of 0.35 μg/mL between the end of primary series and when the booster dose was given at 11 months of age [3].These findings suggest that country-specific serotype-specific immunogenicity should be considered when deciding the appropriate PCV formulation and schedule to implement.

Apart from IPD, non-invasive pneumococcal disease, such as acute otitis media (AOM) and community-acquired pneumonia (CAP) cause significant childhood and adult morbidity. A study by De Gier et al. examined the effect of PCV10 and PCV13 on nasopharyngeal carriage of pneumococcus and non-typeable *Haemophilus influenza* (NTHi), which are a prerequisite for the development of AOM [4]. The authors found that PCV10 does not result in the reduction of NTHi carriage and is associated with higher pneumococcal carriage compared to PCV13. Olasupo et al. addressed the relative clinical and cost burden of CAP among older adults in the US, which could be limited by immunization of older adults against pneumococcus according to CDC guidance [5].

A reoccurring issue since the licensure of PCV13 in 2010 is the lower immunogenicity and/or efficacy for serotype 3 compared to the other vaccine serotypes. A communication by Linley et al. summarizes the efficacy data from seven countries and discusses the limited reliability of serotype 3 antibody measurements using the Luminex assay platform [6]. The authors conclude that further research is required to understand the immune response to serotype 3 in order to improve future vaccine formulations that include this serotype.

New generation vaccines including protein-based vaccines and whole cell vaccines may alleviate some of the limitations of the currently used PCVs, particularly the issue of serotype replacement. A review article by Lagousi et al. discusses the potential use of distinct pneumococcal protein fragments as novel vaccine antigens with special focus on new delivery system technologies, such as the conjugation to Toll-like receptors and reformulation into nanoparticles in order to enhance antigen immunogenicity [7].

The field of pneumococcal vaccines is a constantly changing one with many exciting developments anticipated over the coming years in relation to novel vaccines, abbreviated vaccination schedules and identification of novel immunological markers of vaccine-induced protection. It has been our pleasure to edit this Special Issue on the current state-of-play in relation to the use of pneumococcal vaccines.
